# Micronodular thymoma with lymphoid stroma diagnosed 10 years after the first operation: a case report

**DOI:** 10.1186/s13256-019-2006-y

**Published:** 2019-03-16

**Authors:** Yasunori Kaminuma, Masayuki Tanahashi, Haruhiro Yukiue, Eriko Suzuki, Naoko Yoshii, Toshio Fujino, Hiroshi Ogawa, Hiroshi Niwa

**Affiliations:** 1 Division of Thoracic Surgery, Respiratory Disease Center, Seirei Mikatahara General Hospital, 3453, Mikatahara-cho, Kita-ku Hamamatsu, Shizuoka Japan; 2 Department of Pathology, Seirei Mikatahara General Hospital, 3453, Mikatahara-cho, Kita-ku Hamamatsu, Shizuoka Japan

**Keywords:** Micronodular thymoma with lymphoid stroma, Thymic cyst, Incomplete resection, Local recurrence

## Abstract

**Introduction:**

Micronodular thymoma with lymphoid stroma is a rare subtype of thymoma. Here we report a case of micronodular thymoma with lymphoid stroma that was completely resected after incomplete resection 10 years earlier.

**Case presentation:**

A 70-year-old Japanese woman who had undergone resection for a thymic cyst 10 years earlier was found to have a solid nodule with a multilocular lesion at the site of the previous operation. We suspected that the tumor was a malignant tumor and performed trans-sternal radical thymectomy and diagnosed the lesion as micronodular thymoma with lymphoid stroma pathologically. When we reassessed the thymic cyst that had been resected 10 years earlier, a few lesions of micronodular thymoma with lymphoid stroma were found in the cyst wall. Based on these findings, we concluded that only the cystic component of micronodular thymoma with lymphoid stroma had been removed, and that the residual lesion grew locally over the next 10 years before being completely resected by reoperation.

**Conclusion:**

We experienced an unusual case of micronodular thymoma with lymphoid stroma, which is a rare subtype of thymoma. Greater care should be taken to exclude a thymoma with a cystic lesion, even if a thymic cyst is strongly suspected on computed tomography and magnetic resonance imaging.

## Introduction

Micronodular thymoma with lymphoid stroma (MNT) is a rare subtype of thymoma [1]. Only a few cases of MNT have been reported, especially concerning the long-term prognosis. MNT is a slow-growing neoplasm and no cases of recurrence after operation, distant metastasis, or cancer death with this entity have been reported. Therefore, additional treatment is thought to be unnecessary after complete resection. Here we report a case of MNT that was incompletely resected 10 years earlier and was completely resected after it began to regrow. To the best of our knowledge, this is the first report of a case of local recurrence of MNT. Furthermore, the tumor was misdiagnosed as a thymic cyst at the previous operation 10 years earlier, so it is necessary to consider a thymoma with cystic lesion even if thymic cyst is highly suspected.

## Case presentation

**Fig. 1:**
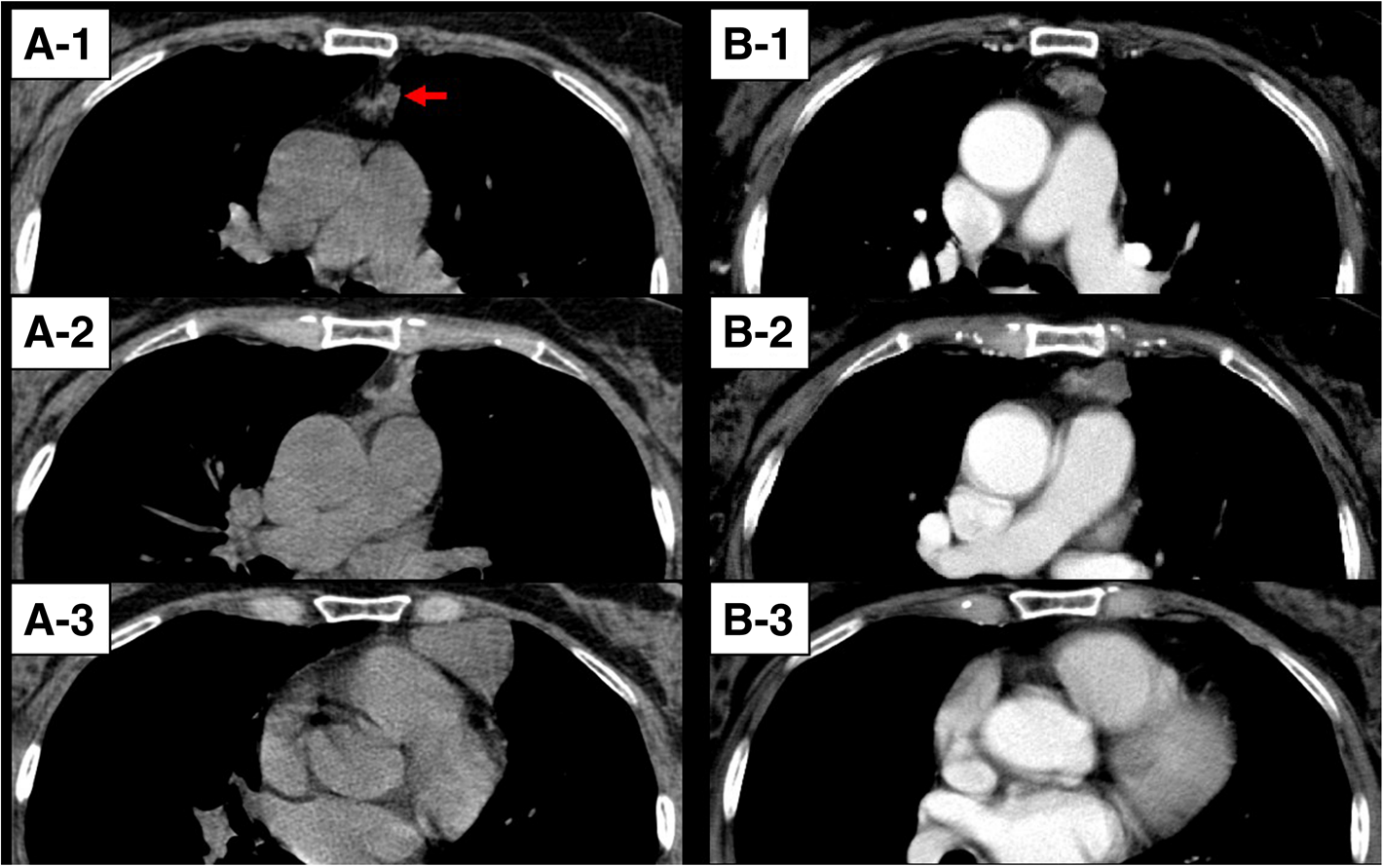
Computed tomography findings. **a** Retrospectively, chest computed tomography image which was taken before the previous operation shows not only cyst which was resected but also a small nodule (*arrow*) which was not resected at the anterior mediastinum. **b** Chest computed tomography imaging which was taken before the present operation shows heterogeneously enhancing mass at the anterior mediastinum. The mass seems to grow from the previously existed small nodule

A 70-year-old Japanese woman who had undergone resection of a thymic cyst by video-assisted thoracic surgery via the left thorax 10 years earlier was found to have a solid nodule with a multilocular lesion at the site of the previous operation. She had no remarkable medical history aside from the tumor and was not taking any medications. She had no tobacco smoking or drinking of alcohol habits, or significant family history. In addition, she was a clerical worker with no exposure to cancer-causing agents. Her physical examination and laboratory findings, including tumor markers, were within normal ranges. Contrast-enhanced computed tomography (CT) of her chest revealed a solid nodule with a multilocular lesion (30 × 30 × 15 mm) at the anterior mediastinum. The solid component was heterogeneously enhanced, and the cystic component was not (Fig. 1b). Retrospectively, the CT findings obtained before the previous operation showed a thymic cyst and a small nodule in the cranial section of the cyst (Fig. 1a). Only the cyst was resected with the partial thymus, and the nodule remained after the previous operation 10 years earlier. Magnetic resonance imaging (MRI) revealed that the solid component was iso-intense on T1-weighted imaging (T1WI) and T2-weighted imaging (T2WI), while the cystic component was iso-intense on T1WI and highly intense on T2WI; there was no evidence of invasion to the surrounding organs.

**Fig. 2:**
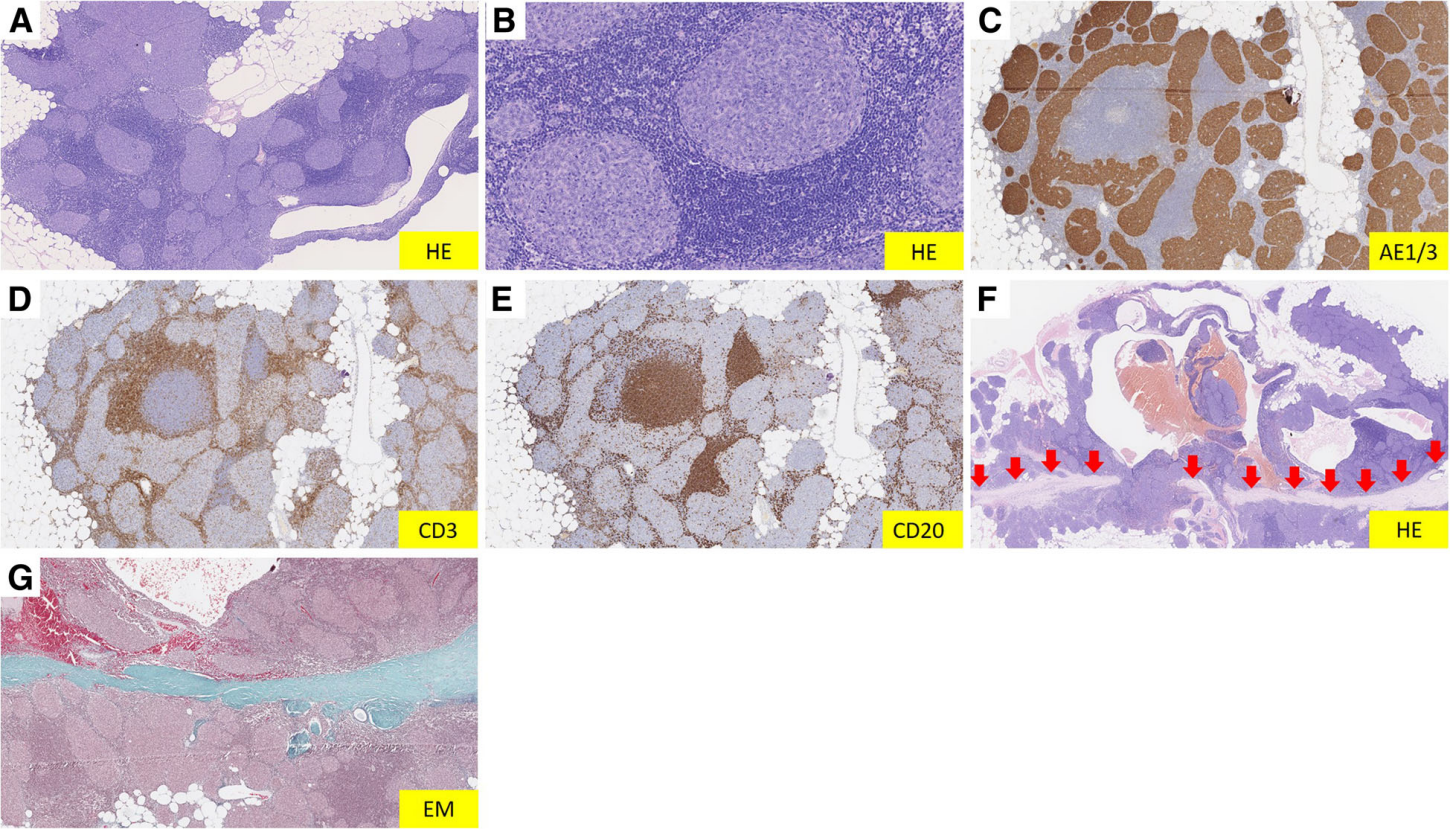
Histological finding of thymic tumor resected at the present operation. **a** Low-magnification image shows small epithelial tumor nodules are separated by abundant lymphoid stroma (hematoxylin and eosin stain). **b** High-magnification image shows the nodules consist of spindle and oval epithelial tumor cells. **c** Epithelial tumor nodules are well stained by cytokeratin staining (AE1/3). **d**, **e** Most lymphocytes had positive immunostaining for CD3 (= T cell) or CD20 (= B cell), and scattered lymphoid follicles were positive for CD20. **f** Linear connective tissue (*arrow*) is found in the center of the tumor (hematoxylin and eosin stain). **g** The connective tissue is stained blue-green by elastica-Masson staining. The tissue is identified as collagenous tissue, and assessed as scar tissue from the previous operation. *EM* elastica-Masson, *HE* hematoxylin and eosin stain

**Fig. 3:**
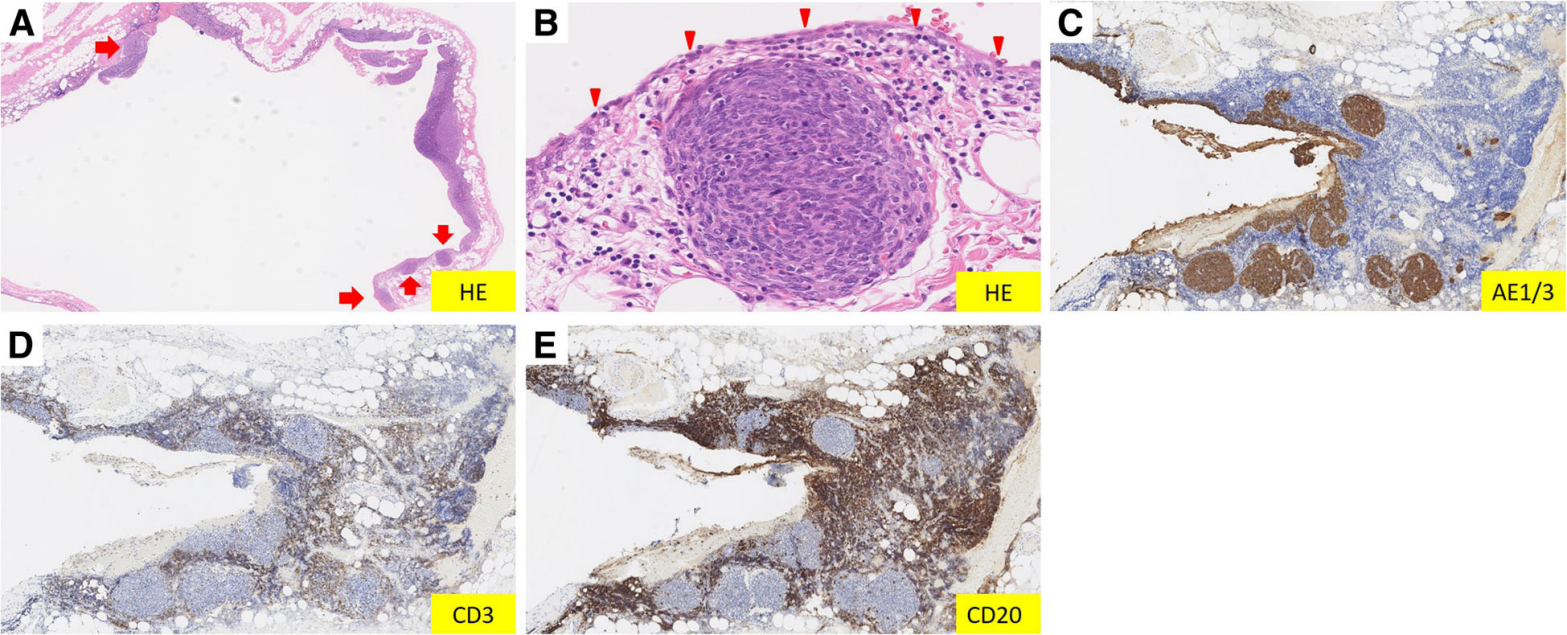
Histological finding of the cyst resected at the previous operation. **a**, **b** Small epithelial tumor nodules and lymphoid stroma which resemble Fig. 2a, b exist in the cyst wall (*arrow*). There is a layer of squamous and cuboidal epithelial cells that line the wall (*arrowhead*). **c**–**e** The immunostaining patterns of the secondary resected thymic tumor and the previously resected cyst are the same, so the cyst is re-diagnosed as cystic lesion of micronodular thymoma with lymphoid stroma. *HE* hematoxylin and eosin stain

Given the possibility that the tumor was malignant (for example, thymoma or thymic carcinoma), we performed trans-sternal radical thymectomy. The tumor was located at the left inferior pole of the thymus, and the cystic component contained serous fluid. There was no dissemination or adhesion. A pathological examination showed that the tumor size was 3.7 × 2.5 × 1.0 cm. On microscopic examination, the tumor was composed of small nodules and abundant lymphoid stroma (Fig. 2a, b). The nodule consisted of short spindle-shaped and oval-shaped epithelial cells that resembled type A thymoma. The lymphoid stroma was composed of normal lymphocytes, and normal lymphocytes formed lymphoid follicles partially. On immunohistochemistry, the epithelial tumor nodules were positive for cytokeratin (AE1/3), and the lymphoid stroma was positive for CD3 and CD20 (Fig. 2c–e). Furthermore, there was linear connective tissue in another slice of the tumor that was probably a scar from the previous operation (Fig. 2f–h). Reassessment of the thymic cyst resected 10 years earlier revealed a few of the same nodules and lymphoid stroma in the wall of the cyst. The nodule and stroma showed the same immunohistochemical staining pattern as the present case (Fig. 3). This indicated that only the cystic component of MNT was removed at the previous operation, and the residual lesion grew over the subsequent 10 years.

The residual lesion was completely removed with the thymus, and there was no recurrence at 2 years after the operation.

## Discussion

This is a case of MNT that had been misdiagnosed as a thymic cyst at a previous operation 10 years earlier and was completely resected after local recurrence. To the best of our knowledge, this is the first report of recurrence, and no previous reports of MNT have included a follow-up exceeding 10 years.

First reported in 1999 [2], MNT is a rare subtype of thymoma, accounting for almost 1% of cases [1]. The median age of patients is 64, and most are over 40 years of age. MNT is more common in men than women and the male-to-female ratio is 1.3 [3]. Paraneoplastic syndrome is not more frequently associated with MNT than with other types of thymoma. For example, 5% of patients with MNT develop myasthenia gravis, while 9% of them develop other autoimmune diseases (for example, pure red cell aplasia). Most patients are asymptomatic, and half have cystic lesions on imaging [1, 3]. In histology, MNT is defined as having short spindle-shaped and oval-shaped epithelial cells that form small nodules, with many lymphocytes and lymphoid follicles around the nodules [1]. Of note, the lymphocytes and lymphoid follicles are not malignant but are instead thought to be attracted by the immunologic reaction against the nodules [4].

MNT grows slowly, and approximately 95% of cases are pathological stage I or II. There have been no reports of recurrence after operation, distant metastasis, or cancer death due to MNT, so no additional treatment is thought to be necessary after complete resection [1, 5, 6]. In the present case, we diagnosed the cyst resected at the previous operation as only part of the MNT and the remaining lesion continued to grow during the 10-year period after the incomplete resection. The reason for this diagnosis was that the tumor resected during the present operation had linear connective tissue which consisted of scar tissue from the previous operation and the cyst was also found to consist of MNT after a reassessment of the findings. We were able to completely resect the tumor after 10 years because MNT is a low-grade malignancy that regrew only locally.

Regarding the difference between thymic cysts and a cystic lesion of MNT, Suster and Moran reported that a thymic cyst has a layer of epithelial cells that line the wall, while such a layer is absent in the cystic lesions of MNT [2]. However, their findings remain controversial because other experts do not support Suster and Moran’s conclusion [2]. The present case did have a layer of epithelial cells lining the wall, so the presence or absence of this layer cannot be used for the differentiation between thymic cysts and MNT. A careful assessment of the presence of a malignant component in the wall of a cyst is important for avoiding a misdiagnosis.

## Conclusion

This case report describes a case of MNT that regrew after incomplete resection 10 years earlier, and is the first case report to describe recurrence of MNT. MNT is a rare subtype of thymoma, and we could completely resect the tumor at reoperation because it is of low-grade malignancy and is a slow-growing tumor. In some cases, it is difficult to distinguish thymoma with a cystic lesion from a thymic cyst, as in the present patient. However, it is important to assess the presence of a malignant component in the wall of a cyst in order to avoid a misdiagnosis.

## Abbreviations

CT: Computed tomography
MNT: Micronodular thymoma with lymphoid stroma
MRI: Magnetic resonance imaging
T1WI: T1-weighted imaging
T2WI: T2-weighted imaging
